# sRNA Profiler: A User-Focused Interface for Small RNA Mapping and Profiling

**DOI:** 10.3390/cells10071771

**Published:** 2021-07-13

**Authors:** Charith Raj Adkar-Purushothama, Pavithran Sridharan Iyer, Teruo Sano, Jean-Pierre Perreault

**Affiliations:** 1 RNA Group/Groupe ARN, Département de Biochimie, Faculté de Médecine des Sciences de la Santé, Pavillon de Recherche Appliquée au Cancer, Université de Sherbrooke, 3201 Rue Jean-Mignault, Sherbrooke, QC J1E 4K8, Canada; 2 Département de Physique, Université de Sherbrooke, 2500 Boul. Université, Sherbrooke, QC J1K 2R1, Canada; pavithran.iyer@uwaterloo.ca; 3 Faculty of Agriculture and Life Science, Hirosaki University, Bunkyo-cho 3, Hirosaki 036-8561, Japan; sano@hirosaki-u.ac.jp

**Keywords:** viroid, RNA silencing, small RNA, vd-sRNA, deep-sequencing, sRNA profiling

## Abstract

Viroids are circular, highly structured, single-stranded, non-coding RNA pathogens known to infect and cause disease in several plant species. They are known to trigger the host plant’s RNA silencing machinery. The detection of viroid-derived small RNAs (vd-sRNA) in viroid-infected host plants opened a new avenue of study in host–viroid pathogenicity. Since then, several viroid research groups have studied the vd-sRNA retrieved from different host–viroid combinations. Such studies require the segregation of 21- to 24-nucleotide long small RNAs (sRNA) from a deep-sequencing databank, followed by separating the vd-sRNA from any sRNA within this group that showed sequence similarity with either the genomic or the antigenomic strands of the viroid. Such mapped vd-sRNAs are then profiled on both the viroid’s genomic and antigenomic strands for visualization. Although several commercial interfaces are currently available for this purpose, they are all programmed for linear RNA molecules. Hence, viroid researchers must develop a computer program that accommodates the sRNAs derived from the circular viroid genome. This is a laborious process, and consequently, it often creates a bottleneck for biologists. In order to overcome this constraint, and to help the research community in general, in this study, a python-based pattern matching interface was developed so as to be able to both profile and map sRNAs on a circular genome. A “matching tolerance” feature has been included in the program, thus permitting the mapping of the sRNAs derived from the quasi-species. Additionally, the “topology” feature allows the researcher to profile sRNA derived from both linear and circular RNA molecules. The efficiency of the program was tested using previously reported deep-sequencing data obtained from two independent studies. Clearly, this novel software should be a key tool with which to both evaluate the production of sRNA and to profile them on their target RNA species, irrespective of the topology of the target RNA molecule.

## 1. Introduction

Viroids are both the simplest and the smallest known plant pathogens. They consist of a circular, single-stranded, non-coding RNA genome of 246–434 nucleotides (nt) in length [1]. Since viroids do not encode for any known peptides, they rely entirely on their sequence, structure and host factors in order to replicate and induce disease symptoms in their host plants. Upon infection, they induce a wide array of symptoms in their host plants, such as leaf epinasty, leaf distortion, stunting and flower distortion [2,3,4]. Due to both their highly base-paired secondary structures and their RNA-RNA mode of replication, viroids are both inducers and targets of RNA silencing [5].

RNA silencing (RNA interference, [RNAi]) is a multi-layer defense system which protects plants from invading RNA pathogens such as viruses and viroids [6]. Silencing is triggered by the processing of either a double-stranded or a highly structured RNA by the host’s RNase III-type ribonucleases (i.e., DICER or DICER-LIKE), resulting in the production of small interference RNAs (siRNAs) of 21–24-nts in length [7]. In 2001, two research groups independently reported, by RNA gel blot assays, the presence of viroid-derived small RNAs (vd-sRNA) in potato spindle tuber viroid (PSTVd)-infected plants [8,9], suggesting that viroids are the targets of RNA silencing. The introduction of next-generation sequencing (NGS) technology permitted large-scale studies on the accumulation of such vd-sRNAs in viroid-infected plants [10,11,12,13]. The role of vd-sRNAs in both the RNAi-mediated down-regulation of the host’s transcripts and symptom induction has been explored using different host–viroid combinations [14,15,16,17,18]. This has been reviewed elsewhere [19].

Viroid researchers extensively use NGS to study host–viroid interactions, focusing on vd-sRNA production [20]. The profiling of vd-sRNAs on both the genomic and antigenomic strands of a viroid yields several types of information, including, but not limited to: (i) the proportions of vd-sRNAs produced from the genomic and the antigenomic strands of the viroid, respectively; (ii) the distribution of vd-sRNAs on both the genomic and the antigenomic strands; and, (iii) the regions of the viroid that are either susceptible to or resist the host’s RNA silencing machinery. Such information could be used to understand host–viroid species relationships [11,13,21], viroid quasi-species [22,23] and to develop viroid resistant plants [24,25]. First and foremost, in order to obtain all of this information, mapping of deep-sequenced vd-sRNAs is needed on both the viroid genomic and antigenomic sequences. The biggest hurdle for such studies is the lack of software for mapping the sRNA on circular genomes, such as those of viroids. Although several commercial platforms are available, they are all programmed for linear DNA or RNA molecules. Consequently, either viroid researchers develop their own interface, or they depend on the work of bioinformaticians in order to progress their studies.

The mapping of a vd-sRNA to a target viroid’s genomic and/or antigenomic strand sequence is innately tied to the classic example of determining the number of pattern matchings. In computer science, pattern matching is the process of checking a given sequence of characters (in the present scenario, the nucleotides) that exists among the provided data. In other words, given two strings α and β, whether string β occurs as a sub-string of α. Knuth, Moris and Pratt first addressed the concept of pattern matching in 1970 [26]. Since then, many solutions to this problem have been proposed. Pattern matching is extensively used in bioinformatics in order to determine the sequence similarity between the subject and the query [27].

In this study, python language was used to write the pattern matching program so as to provide a user-friendly and efficient bioinformatic tool to viroid researchers. Python is one of the few computer languages that is easy to both read and implement. Additionally, it is an open-source language and runs on different platforms, such as Mac, Windows and Unix. Here, the development of an interface, which was initially tested on an illustrative genomic sequence and sRNAs and then applied to the previously reported NGS data, is reported.

## 2. Materials and Methods

### 2.1. Setting the Parameters for Pattern Matching

The counting version of standard pattern matching is a problem of enumerating all of the occurrences of the string β in the string α [28,29]. Computational biology provides an archetypal context for counting problems of this sort [27,30,31]. For example, if β is considered as being an sRNA and α as being a viroid genome, the real-world problem is computing the number of bindings of a vd-sRNA with the viroid genome. When an sRNA nucleotide sequence is represented by a string β, and the viroid genome by a string α, computing the number of bindings of the sRNA sequence immediately admits the counting form of the standard pattern matching problem. Furthermore, one can safely assume that α and β are drawn from the alphabet $\left\{A,T,G,C\right\}$. We want to solve several instances of the above counting problem, each corresponding to a different viroid RNA nucleotide α. Following is the precise statement of the problem.

**Definition** **1.** *Viroid binding problem.*

*Given the following:*

- *Set* $S_{1}$*of N strings, where each string* $s_{1}\in S_{1}$*has length* k, *sampled from* $\left\{A,T,G,C\right\}$;
- *String* $s_{2}$*of length m, sampled from the alphabet* $\left\{A,T,G,C\right\}$; and,
- *Function*η*that maps strings to* $\left\{0,1\right\}$*to identify a matching between two strings, as follows. For two equal-length strings* α*and* β, $\eta \left(\alpha ,\;\beta \right)=\;1$*if and only if* α*and* β match.

*Compute the size of the sets* $\chi_{i}$*for each* 1≤i≤m, where:

(1) $$\chi_{i}=\left\{\;j:\;1\leq j\leq m,\;\eta \left(\alpha_{j,\;j+k},\;\beta \right)=1\right\}$$

The definition of η is intentionally unspecified so as to accommodate various scenarios in the present work. That said, η is used in four different ways that can be divided into two categories: (i) the matching of β in the forward and reverse directions with respect to α; (ii) the linear or circular topology of α.

The first set of variations occurs from choosing an alignment of the viroid genome relative to the sRNA. While the natural alignment, which is called the forward matching, coincides with the definition in “Definition 1,” its counterpart is referred to as the reverse matching set. The forward and reverse cases differ in their rules for deciding when two equal-length strings α and  β, sampled from the same alphabet, are identical. The forward matching setting adopts the natural definition where $\eta \left(\alpha ,\beta \right)=1$ if and only if $\alpha_{i}=\beta_{i}$, for all 1≤i≤n, where n is the length of α. On the contrary, the reverse matching setting places a non-standard requirement: $\eta \left(\alpha ,\beta \right)=1$ if and only if $\alpha_{i}=\bar{\beta_{n-i}}$, for all 1≤i≤n. Moving forward, only the case of forward matching is discussed, as reverse matching adopts the same discussion of forward matching, without affecting the conclusion.

Setting the topology is crucial in determining the boundaries for a string that is able to differentiate the mapping of sRNA derived from linear and circular genomes. The case of linear topology (used for sRNA derived from a linear genome), which is referred to as the linear alignment, refers to an α with a finite boundary, that is to say, a length k string α with $\alpha_{j+1}$ following $\alpha_{j}$ for 1≤j≤k−1 and with no character following $\alpha_{k}$. On the contrary, the case of the circular topology (used for sRNAs derived from a circular genome), which is referred to as the circular alignment, is characterized by a gene with periodic boundary conditions, that is to say a length k string α with the character $\alpha_{\left(j+1\right)\;mod\;k}$ following the character $\alpha_{j\;mod\;k}$ for 1≤j≤k. Clearly, the matching problem on a circular aligned gene trivially reduces to the linear alignment case. This can be realized by appending the gene string α with the length  k substring of α, starting at position 1. Consequently, no conceptual distinction will be attributed to this setting, and only the solution for the linear alignment will be analyzed.

### 2.2. Finding the Occurrence of β in α

This section presents the solution to forward matching with linear alignment for the pattern matching problem defined in Definition 1. First of all, the following useful notations need to be introduced.

The *length* of a string α is simply the number of characters in α. Oftentimes, $\left|\alpha \right|$ is used to denote the size of α.

The *Hamming weight* of a binary sequence b is the number of 1s in the sequence. Oftentimes, wt(b) is used to denote the Hamming weight of b.

A *naive* solution to the pattern matching problem entails a systematic search for a match between every sequence in $S_{1}$ to every substring of $s_{2}$. In other words, it makes $\left|S_{1}\right|$ call to an elementary routine which compares two strings, $s_{1}$ and $s_{2}$, in order to identify the number of times $s_{2}$ occurs in $s_{1}$. Consequently, the time complexity of the naive solution is $O\left(n\;k^{2}\right)$. A solution that outperforms the naive one is the goal. This is achieved by mapping the pattern matching problem to a similar size matrix multiplication problem. The latter can be solved efficiently by vectorizing individual operations.

The first ingredient of this mapping is encoding each character in $\left\{A,T,G,C\right\}$ into a unique binary sequence of 4 bits.

(2) A→1000, T→0100, G→0010, C→0001

The above encoding naturally implies an encoding for strings composed of the characters from $\left\{A,T,G,C\right\}$ by simply concatenating the binary sequences corresponding to the individual alphabets in the string. Likewise, a nucleotide sequence α of length k is identified by a binary sequence of 4k bits, and is denoted by $b\left(\alpha \right)$.

It should be noticed that distinct characters in Equation (2) correspond to orthogonal binary sequences. Therefore, two nucleotide strings α and β, each of length k, match exactly if and only if the number of ones in the dot product $b\left(\alpha \right)\cdot b\left(\beta \right)$ is equal to k, that is to say, $\mathrm{wt}\left(b\left(\alpha \right)\cdot b\left(\beta \right)\right)$. A similar observation can be made in order to account for δ mismatches, wherein the number of ones in $b\left(\alpha \right)\cdot b\left(\beta \right)$ should be at least k−δ. To summarize, two length k strings α and β match approximately with at most δ mismatches if and only if the Hamming weight of $b\left(\alpha \right)\cdot b\left(\beta \right)$ is at most k−δ.

The above describes a method with which to check for a match between two strings of equal length. This can be readily extended to strings of unequal lengths, such as when $\left|\alpha \right|=n$ and $\left|\beta \right|=k$ for k<n. In order to do this, note that β can be found in α if and only if there are k consecutive characters in α that match with β. In other words, $\beta =\alpha_{j,j+k}$ for some $j\in \left[1,\;n-k\right]$. Furthermore, there are exactly n−k substrings of α that can be formed by taking n consecutive characters, described by the set $\left\{\alpha_{1,k},\;\ldots ,\;\alpha_{n-k,\;n}\right\}$. Each of these substrings $\alpha_{j,j+k}$ can be encoded into a binary sequence $b\left(\alpha_{j,j+k}\right)$ having 4k bits. The substrings $\alpha_{j,j+k}$ that match with β will can be identified by checking if $\mathrm{wt}\left(b\left(\alpha_{j,\;j+k}\right)\cdot b\left(\beta \right)\right)$ is at least k−δ.

Lastly, we can combine the tests for matchings between each example of $\alpha_{j,j+k}$ for 1≤j≤n−k and β, into a single condition. Defining $B\left(\alpha \right)$ to be a $k\times \left(n-k\right)$ matrix whose k columns are $b(\alpha_{j,j+k}),\;\ldots ,\;b\left(\alpha_{n-k,\;n}\right)$, it turns out that the dot product $v=b\left(\beta \right)\cdot B$ is a vector of size n−k, whose i−th component $v_{i}$ satisfies $v_{i}\geq k-\delta$ if and only if the substring $\alpha_{j,j+k}$ matches with β at all locations except for at most δ of them. In other words, the number of occurrences of β in α is determined by the number of entries in $b\left(\beta \right)\cdot B$, whose value is at least k−δ. This inference forms the backbone of the solution to the underlying pattern matching problem.

Summarizing the solution to the forward matching problem with the linear alignment presented in Definition 1, and recalling that the problem here is defined by a set of sRNA nucleotides $S_{1}$ and a gene $s_{2}$, the encoding in Equation (1) leads to two key observations:

$S_{1}$ is mapped to an $\left(N\times k\right)$ binary matrix $B_{1}$, whose i-th row denotes the binary encoding of the i-th nucleotide in the sRNA pool.

$s_{2}$ is mapped to a $\left(k\times n\right)$ binary matrix $B_{2}$, whose i-th column denotes the binary encoding of the length k substring of $s_{2}$, starting at position i.

These observations imply that the dot product $M=B_{1}\cdot B_{2}$ yields an (N×n) binary matrix, where $M_{i,j}$ is equal to 1 if and only if the i-th sRNA nucleotide binds to the gene at position j. Finally, the sum of all the rows of M yields a vector V of size n, where $V_{j}=\sum_{i=1}^{N}M_{i,j}.$ Clearly, $V_{j}$ is nothing but the number of sRNA nucleotides in $S_{1}$, which binds to the gene $s_{2}$ at location j. In other words, Equation (1) takes the form $V_{j}=\chi_{j}$. This completely specifies the solution to the pattern matching problem. Casting the pattern matching problem as matrix multiplication is critical [32] to its low runtimes for large number of sRNA nucleotides.

### 2.3. Profiling of vd-sRNA on Both the Viroid Genomic and Antigenomic Strands

The key input data for the program are the genome sequence, specified in a text file without line-breaks, and the pool of vd-sRNA, specified in a text file wherein each new line identifies a nucleotide sequence composed of “A,” “T,” “G” and “C” characters. If there are any nucleotide sequences that contain an “N,” they are ignored. In order to accommodate various formats, the program accepts a whole number which specifies the number of lines to skip before reading every nucleotide sequence in the pool file. Additional settings for the matching problem include: (i) tolerance, the maximum number of mismatches allowed in order to accommodate the vd-sRNAs derived from the quasi-species and (ii) the topology of the gene, that is to say a Boolean expression that takes the value 0 for linear matching and 1 for circular matching. For example, “gene.txt pool.txt 2 1 1” is a complete input specification indicating the problem of computing bindings in the circular topology condition, while allowing for, at most, one mismatch between the sequence in gene.txt with those in the pool.txt. All such instances of the matching problem can be gathered in a text file and placed in “vbind/data/input.” The output format for the solution of the matching problem is a matrix, each row of which corresponds to a unique length of vd-sRNA sequences in the pool. Each row is of a length equal to the gene sequence, where its i-th column specifies the number of matchings amongst sequences of a given length in the pool, with the sequence of the gene starting at position 1.

### 2.4. Viroid Small RNA Data

Two previously published sRNA datasets obtained from tomato plants infected with variants of PSTVd were used in this study. Specifically, the total sRNA sequences obtained from tomato plants infected with either PSTVd-I (GenBank Acc. No.: AY937179) or PSTVd-RG1 (GenBank Acc. No.: U23058) having the GEO Acc. No. GSM1695657 and GSM1717894, respectively [16,33], were used.

### 2.5. Software Accessibility and Instructions

The software is available at https://github.com/paviudes/vbind (accessed on 5 July 2021). Example input and running instructions as shown in data/input/example.txt. Furthermore, for developers in Python, the key functions in the software that help solve the viroid binding problem as well as the visualization tools are available as a Python package in the PyPI repository: https://pypi.org/project/vbind/ (accessed on 5 July 2021). 

## 3. Results

### 3.1. Overview of sRNA Mapping and Profiling

**Figure 1:**
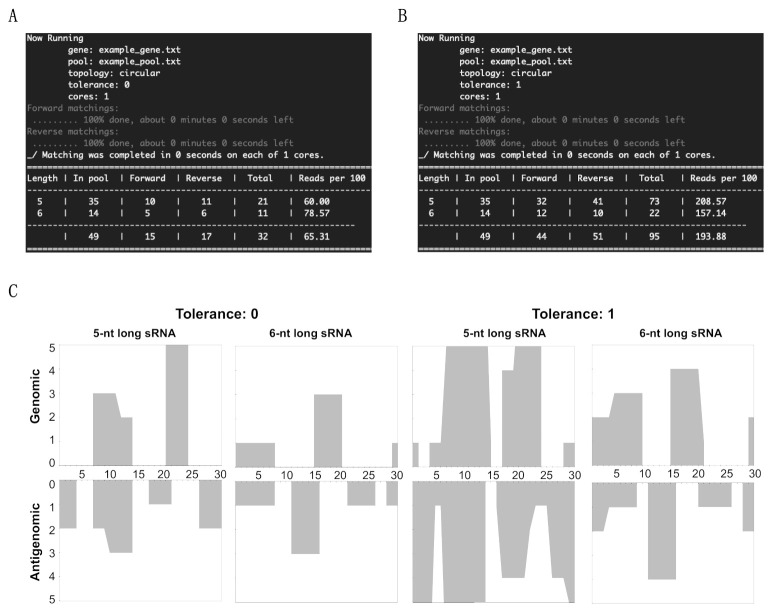
sRNA profiler data output for the test sample. (**A**) Summary of the mapping data obtained from “0” tolerance and (**B**) “1” tolerance levels. (**C**) Profiling of the mapped sRNAs on the 30-nt long circular test genome at both the “0” and the “1” tolerance levels for 5- and 6-nts long sRNAs.

In order to verify the effectiveness of this new tool, a 30-nt long DNA string of (5′- GCT TCA GGG ATC CCC GGG GAA ACC TGG TCG-3′) was used as the “genome,” that is to say that it was the equivalent of the viroid genomic strand. From this DNA string, a random pool of sRNAs was prepared such that the pool contains at least one non-matching sRNA, one genomic sRNA and one antigenomic sRNA of both 5- and 6-nts in length. Additionally, a couple of sRNAs were taken between positions 27 and 5 of the DNA string in order to imitate the sRNA derived from the circular genomic strand (Table 1). This pool is equivalent to the NGS data of the sRNAs. The results obtained by running the patterning matching program in order to identify the matches between the sRNAs in the pool and the genome strand are summarized by the results visible on the screen (Figure 1, panels A to D). The data obtained include the name of the file containing the gene sequence, the sRNA pool, the topology, the tolerance level, the cores used for the mapping, a table summarizing the total number of sRNAs of the different lengths, the number of sRNAs matching in the forward direction, the number of sRNAs matching in the reverse direction, the total number of sRNA matching and the percentage of sRNAs mapped for the given sRNA species. The details of each sRNA sequence that occurred in the pool are presented in Figure S1, as they were generated by the sRNA profiler.

In order to profile the sRNA on both the genomic and antigenomic strands of the viroid genome, the above matched sRNAs were run on the genome strand. These results were visualized using the post-processing tools available from by executing “./analyze.sh.” As presented in Figure 1C, the *X*-axis indicates the length of the viroid RNA, while the *Y*-axis shows the number of matching sRNAs. By testing the above example, it is clear that the tool developed here can detect the total sRNA count, and the sRNAs matching both the genomic and the antigenomic strands, thus addressing the fundamental questions of mapping the sRNAs from the NGS data on a given genome sequence. Furthermore, profiling of the mapped sRNAs on the illustration DNA strand shows exactly how the sRNA profiler developed in this study could be used to analyze vd-sRNAs obtained from viroid-infected plants.

### 3.2. Mapping of sRNAs Obtained from PSTVd-I-Infected Plants

In order to map and profile the sRNAs on both the genomic and antigenomic viroid RNA strands, an sRNA pool of fragments 15- to 37-nts in length obtained from PSTVd-I-infected tomato plants was retrieved from GEO. The dataset was processed with the 359-nts long PSTVd-I genome sequence with zero tolerance, so as to segregate the genomic and antigenomic matching vd-sRNAs.

As vd-sRNAs of 21- to 24-nts in length are the those of interest, the matchings obtained for these sizes are presented in Table 2. Out of 4,316,543 sRNAs of 21- to 24-nts, a total of 488,176 vd-sRNAs showed 100% sequence similarity with the PSTVd-I sequence, accounting for 11.3% of the total recovered sRNAs. More specifically, 380,731 (8.8%) and 107,445 (2.5%) vd-sRNAs of 21- to 24-nts were derived from the genomic and antigenomic strands of PSTVd-I, respectively. In order to obtain a better picture of the expression levels of the individual-sized vd-sRNAs, the obtained vd-sRNAs were normalized per one million reads. The normalization feature is incorporated into the software’s post-processing toolkit. It can be invoked as a separate feature while executing “./analyze.sh” as well as an addon feature while plotting. Detailed analysis showed that 22-nts long sRNAs had a maximum number of vd-sRNAs (34.3%), whereas the least expressed vd-sRNAs were those 24-nts in length (1.2%). Interestingly, the vd-sRNAs based on individual sizes revealed that the genomic strand produced more 22-nts sRNAs (29.3%), while the antigenomic strand produced more 21-nts sRNAs (7.9%). Overall, more genomic strand-derived sRNAs were recovered as compared to the antigenomic strand in the case of PSTVd-I. In other words, (+) vd-sRNAs were expressed 3.5 times more than (−) vd-sRNAs. This can be attributed to the higher recovery of the genomic strand of the viroid in infected plants, as described elsewhere [34]. These data are in agreement with the previous report, where the same sRNA pool was used for the analysis [16], confirming the reproducibility of the new software.

### 3.3. Profiling of the Mapped sRNAs on PSTVd-I

**Figure 2:**
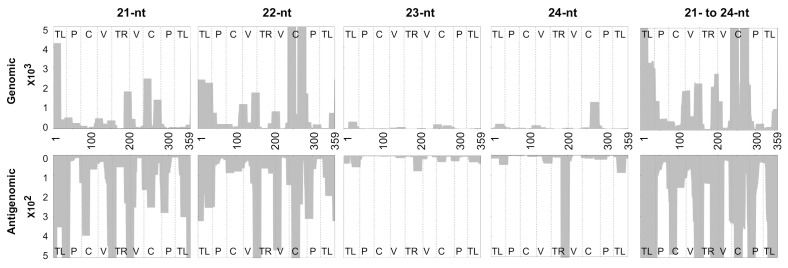
Profiling of the vd-sRNAs recovered from PSTVd-I-infected tomato plants at zero mismatches. Sequence profiles of the (+) and the (−) PSTVd-sRNA populations recovered from the leaf tissues of infected tomato plants. The data were normalized to reads per million. In the figure, different structural regions of PSTVd are delimited by dashed lines (from left to right, TL, terminal left; P, pathogenicity; C, central; V, variable; and TR, terminal right).

In order to examine the regions of PSTVd that produced the most vd-sRNA, the above-mapped vd-sRNAs were plotted on both the genomic and the antigenomic strands of PSTVd-I. Hence, each 21- to 24-nts long vd-sRNA, and the cumulative 21- to 24-nts long vd-sRNAs, were profiled on PSTVd-I (Figure 2). The data presented here clearly identify the regions that produce the most vd-sRNA on both the genomic and antigenomic strands. This result is in agreement with a previous report [16], confirming the reproducibility of this novel profiling tool.

### 3.4. Accommodating vd-sRNA of the PSTVd-I Quasi-Species

**Figure 3:**
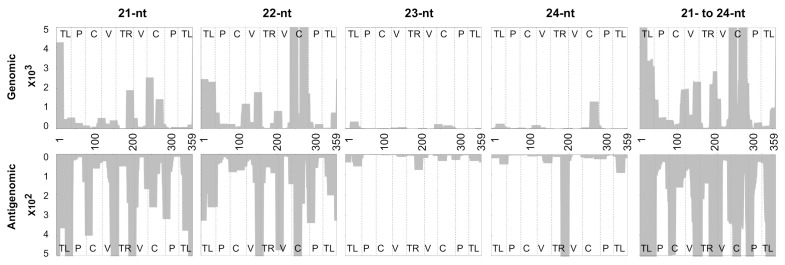
Profiling of the vd-sRNAs recovered from PSTVd-I-infected tomato plants at one mismatch. Sequence profiles of the (+) and the (−) PSTVd-sRNA populations recovered from the leaf tissues of infected tomato plants. The data were normalized to reads per million. In the figure, different structural regions of PSTVd are delimited by dashed lines (from left to right, TL, terminal left; P, pathogenicity; C, central; V, variable; and TR, terminal right).

Viroids are known to form quasi-species in host plants [22,23,35,36,37]. That said, a single sequence type could give rise to several hundred or even thousands of sequence variants in host plants. In order to accommodate the vd-sRNA derived from the PSTVd-I sequence variants, in this study, the strictness of the vd-sRNA mapping against both the genomic and antigenomic strands of PSTVd-I was decreased by allowing for the presence of at least one mismatch. This decrease of the stringency increased the overall matching by 0.4% as compared to that obtained with zero mismatches (also called the “zero tolerance”), more precisely from 11.3 to 11.7% (Table 3). In other words, 113,094 and 117,011 vd-sRNAs of 21- to 24-nts long are present per million sRNAs with zero tolerance and one mismatch, respectively. Allowing for the presence of one mismatch increased the percentage of matching from as low as 0.1% (for 24-nts long vd-sRNA) to as high as 1.1% (for 22-nts long vd-sRNA), as compared to having zero mismatches. However, the overall distribution of (+) vd-sRNA to (−) vd-sRNA remained almost identical to that seen with zero mismatches. Furthermore, as with zero tolerance mapping, one mismatch also had the highest number of genomic derived vd-sRNAs for 22-nts long sRNAs, whereas for the antigenomic derived vd-sRNAs, the 21-nts long ones did. In order to evaluate the regions of PSTVd that produced the most vd-sRNA, the above-mapped vd-sRNAs with one mismatch were plotted on both the genomic and the antigenomic strands of PSTVd-I, as described above (Figure 3).

### 3.5. Evaluating the Mapping Tool Using the vd-sRNAs Obtained from PSTVd-RG1-Infected Plants

In order to increase the confidence in the programing tool, one more sRNA dataset that is publicly available was analyzed. Specifically, the sRNAs sequences obtained from tomato plants infected with the PSTVd-RG1 variant were used. Both PSTVd-I and PSTVd-RG1 possess 359-nts long genomes. PSTVd-I and PSTVd-RG1 have three mismatches in their genomes and they induce intermediate and lethal disease symptoms in tomato cultivar Rutgers (*Solanum lycopersicum* cv Rutgers), respectively [18]. As described earlier, a total of 730,499 sRNAs of 21- to 24-nts in length were mapped on PSTVd-RG1 in both the forward and the reverse directions in order to segregate the genomic (+) and the antigenomic (−) vd-sRNAs at both the zero and one mismatch levels of tolerance. The resulting data were normalized per million reads (Table 4). The results showed that a total of 102,555 vd-sRNAs were recovered per one million reads, and that these contained 88,493 (8.8%) and 14,062 (1.4%) genomic strand- and antigenomic strand-derived vd-sRNAs, respectively, which showed 100% sequence similarity with PSTVd-RG1. This accounted for 10.3% of all sequenced 21- to 24-nts long sRNAs. Out of all 21- to 24-nts long vd-sRNAs, the 21-nts long vd-sRNAs species were the most highly expressed (25.8%), while the 24-nts long vd-sRNAs were the least expressed (1.1%), for both the genomic and the antigenomic strands of PSTVd-RG1.

In order to allow for the vd-sRNAs derived from the sequence variants of PSTVd-RG1, one mismatch was allowed in the mapping. Decreasing the stringency increased the number for vd-sRNAs by 3.3% (from 102,555 per million reads with zero mismatches to 135,654 per million reads with one mismatch: Table 5). This was attributed to the 2.9% and 0.4% increases in the genomic and antigenomic strands’ vd-sRNAs, respectively, as compared to the zero-tolerance mapping. Analyzing the vd-sRNAs derived from both the genomic and the antigenomic strands revealed that genomic strand-derived sRNAs were expressed at least 6.4 times higher than the antigenomic strand-derived sRNAs.

**Figure 4:**
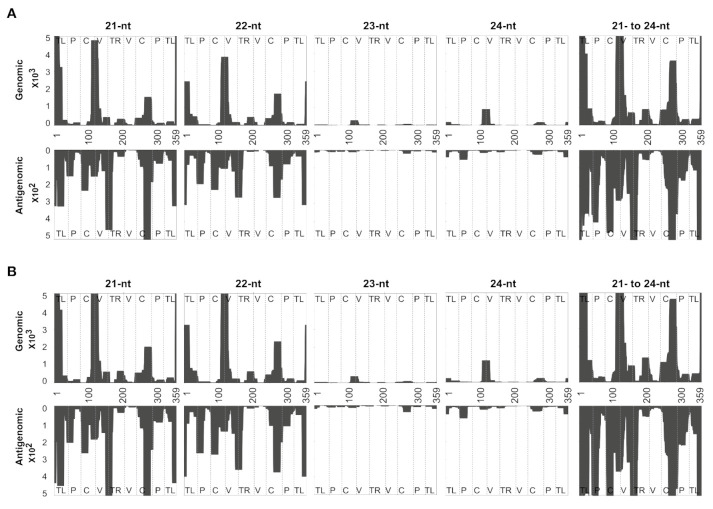
Profiling of the vd-sRNAs recovered from PSTVd-RG1-infected tomato plants at zero and one mismatches. Sequence profiles of the (+) and the (−) PSTVd-sRNA populations recovered from the leaf tissues of infected tomato plants. Panels (**A**) represent the profiles of the (+) and the (−) PSTVd-RG1-derived sRNAs of zero mismatches of sizes 21-, 22-, 23-, 24-nts and of the cumulative of those of 21- to 24-nts; Panels (**B**) represent the profiles of the (+) and the (−) PSTVd-RG1-derived sRNAs of one mismatch of sizes 21-, 22-, 23-, 24-nts and of the cumulative of those of 21- to 24-nts, respectively. The data were normalized to reads per million. In the figure, different structural regions of PSTVd are delimited by dashed lines (from left to right, TL, terminal left; P, pathogenicity; C, central; V, variable; and TR, terminal right).

In order to visualize the regions of PSTVd-RG1 that produced vd-sRNAs, derived from both the genomic and the antigenomic strands, the mapped vd-sRNAs with both zero and one mismatch were plotted on both the genomic and the antigenomic strands of PSTVd-RG1 (Figure 4). The profiles obtained for 21-, 22-, 23-, 24-nts long vd-sRNAs and the cumulative 21- to 24-nts long vd-sRNAs are presented in Figure 4; panels A and B show zero and one mismatch, respectively.

## 4. Discussion

Although viroids are single-stranded, circular RNA molecules, they as both inducers and targets of the host’s RNA silencing machinery (reviewed in [5]) due to the fact that they: (i) possess sequence complementarity and, consequently, form highly base-paired secondary structures; (ii) replicate through either an asymmetric or a symmetric rolling circle mechanism. That said, upon infection, all viroids trigger RNA silencing, and this results in the cleavage of the viroid RNA into sRNAs of 21- to 24-nts in length. The accumulation of these vd-sRNAs has been extensively studied in different host–viroid combinations [10,11,13]. Since viroids are non-coding pathogens, recent works were directed towards understanding the role of such vd-sRNAs in both their pathogenicity and symptom induction [14,15,16,19,21,38,39,40]. Included in these studies was the prediction of the vd-sRNA:target mRNA duplex formation and searching for the number of vd-sRNAs in the viroid-infected plant that could potentially bind to a given target mRNA. For such studies, it is critical to map the vd-sRNAs obtained from NGS data derived from viroid-infected plants on both the viroid’s genomic and antigenomic strands. Profiling of the obtained vd-sRNAs on both the genomic and antigenomic strands of the viroid helps identify which regions of the viroid produce the most vd-sRNA and which the least. This information indirectly suggests which regions of the viroid are either more susceptible or more resistant to host RNA silencing. Furthermore, this type of information helps in the development of RNAi mediated viroid resistant transgenic plants [24]. In addition, the mapping of vd-sRNA and their profiling on both the viroid’s genomic and antigenomic strands are very important in understanding the host–viroid interaction.

Viroids, being circular in nature, require a specific computer program in order to be able to map the sRNAs derived from the junction of first and last nucleotides of the viroid. This feature is currently not available in any commercial software. Here, a sRNA profiler has been developed, more precisely a python-based program that was tailormade to address not only this issue, but also to detect both genomic strand- and antigenomic strand-derived sRNAs. Besides its capabilities for detecting mappings, the tool is attractive because of its efficiency (Table 6). For instance, the sRNA pool of 5.8 million reads can be solved in 30 min using a desktop computer with a single core, whereas when using this new tool, solving 1 million reads requires only approximately 2 min. This gain eliminates the need for the high-end hardware that are often required by commercial programs. Moreover, the fact that sRNA profiler is written in python permits its customization for various studies of both circular and linear RNAs.

The solution to the case of a viroid binding problem, as computed by the sRNA profiler, is represented by two matrices with whole number entries: (i) the solution for the forward matchings; (ii) the solution for the reverse matchings. In each of the two cases, the matchings are specified by matrix $M^{+}$ for the forward matchings and $M^{-}$. for the reverse matchings, of size $\left(L\times n\right)$, where L is the number of distinct sRNA in the pool and m is the length of the gene. Each entry, $M_{l,j}^{+}$ or $M_{l,j}^{-}$, is the number of sRNA sequences in the pool of length indexed by l that have a forward or reverse matching, respectively, with a substring of the gene starting at position j. In addition to solving the viroid bindings problem, the sRNA profiler is capable of a visual representation of the output by profiling the vd-sRNAs on both the genomic and the antigenomic strands of the viroid (Figure 2, Figure 3 and Figure 4). In these figures, the *X*-axis shows the positions on the viroid sequence, the forward matching solutions are represented on the positive *Y*-axis and the reverse matchings on the negative *Y*-axis. For each index i on the *x*-axis, the absolute value on the *Y*-axis for a fixed length l is the maximum number of sequences in the pool that match with the substring of the gene starting at position i. The area under the curve is filled in for visual appeal purposes. It is important to note that the message conveyed in Figure 2 and Figure 4 is identical to that of previous studies [16,33], even though the latter one adopted a slightly different visual representation for the plot. In these earlier studies [16,33], a visual representation was used wherein the height of the curve at a given position i on the *X*-axis denoted the number of vd-sRNA nucleotides that match with a substring containing the character of the gene at position i. This can be derived from the $M^{+},M^{-}$ matrices of the sRNA profiler by the use of a simple postprocessing routine. The software’s post-processing toolkit can normalize the sRNA reads per million.

**Figure 5:**
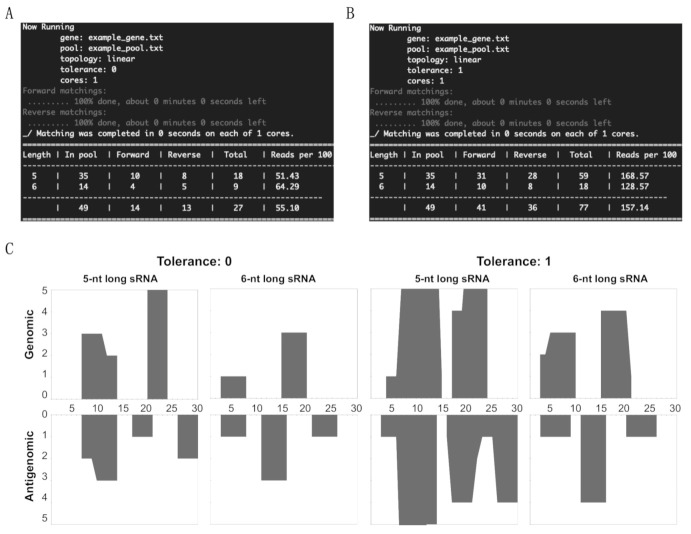
sRNA profiler data output for the linear RNA test sample. (**A**) Summary of the mapping data obtained from “0” tolerance and (**B**) “1” tolerance levels. (**C**) Profiling of the mapped sRNAs on the 30-nt long linear test genome at both the “0” and the “1” tolerance levels for 5- and 6-nts long sRNAs.

To increase the versatility of the sRNA profiler, the mapping of sRNA on circular RNA can be easily converted into linear RNA molecules, such as viral RNA, by changing the topology from circular to linear. To add to the versatility of our sRNA profiler, we have incorporated the ability to map on a linear genome, such as a virus. The choice between a circular or a linear genome is specified in terms of an integer flag—which takes the value 0 to identify a linear genome and the value 1 to identify a circular genome. Figure 5 shows the result of the matching problem on a linear genome with the sample data used for the circular case in Figure 1. The details of each sRNA sequence that occurred in the pool are presented in Figure S2 as they were generated by the sRNA profiler.

In order to provide a user friendly and the efficient bioinformatic tool to viroid researchers, the sRNA profiler was developed by pattern matching using python language. This program seamlessly allows researchers to map and plot sRNAs on their parent circular viroid or linear virus molecule. Choosing the different matching tolerances allows the user to consider and visualize the sRNAs derived from the quasi-species. This program will help researchers in their studies by both evaluating the production of sRNA and their profiling on their target viroid or virus species.

## Figures and Tables

**Table 1:** Summary of the sRNA sequences used in the test run of the sRNA profiler.

sRNA Length	Tolerance: 0		Tolerance: 1		Non-Matching sRNA
	Forward Matching	Reverse Matching	Forward Matching	Reverse Matching	
**5-nt**	GGGAT	ACCAC	ACCCG	AGATT	AGGGA
	ATCCC	CCACG	GGATC	CACGA	CAAGT
	AAACC	CCGTG *	TTGAC *	CCCTG *	GGGGC
**6-nt**	TTCAGG	AGTTCC	ACAAAA	CAGAGG	ACGCAG
	CGGGGA	GTGAAC *	GGCTCA	GTGGAC *	GTAGAT
	CGGCTT *	CCGTGA *	CACCGA *	CCGCGA *	CGGAAA

* sRNAs derived between nucleotide positions 28 and 5, representing the sRNA of a circular genome.

**Table 2:** Summary of the sRNAs identified by NGS from PSTVd-I-inoculated tomato plants cv. Rutgers at zero mismatch with the PSTVd-I genome.

sRNA Length	Total sRNA in the Pool	Tolerance: 0			Normalized Reads (Per Million Reads)							(+):(−) vd-sRNA
		(+) vd-sRNA	(−) vd-sRNA	Total vd-sRNA	Total sRNA	(+) vd-sRNA		(−) vd-sRNA		total vd-sRNA		
						No. of Reads	%	No. of Reads	%	No. of Reads	%	
21-nt	700,131	105,294	55,304	160,598	162,197	24,393	15.0	12,812	7.9	37,205	22.9	1.9
22-nt	839,033	245,515	42,665	288,180	194,376	56,878	29.3	9884	5.1	66,762	34.3	5.8
23-nt	622,373	10,064	3154	13,218	144,183	2331	1.6	731	0.5	3062	2.1	3.2
24-nt	2,155,006	19,858	6322	26,180	499,243	4600	0.9	1465	0.3	6065	1.2	3.1
21–24-nt	4,316,543	380,731	107,445	488,176	1,000,000	88,203	8.8	24,891	2.5	113,094	11.3	3.5

**Table 3:** Summary of the sRNAs identified by NGS from PSTVd-I-inoculated tomato plants cv. Rutgers at one mismatch with the PSTVd-I genome.

sRNA Length	Total sRNA in the Pool	Tolerance: 1			Normalized Reads (Per Million Reads)							(+):(−) vd-sRNA
		(+) vd-sRNA	(−) vd-sRNA	Total vd-sRNA	Total sRNA	(+) vd-sRNA		(−) vd-sRNA		total vd-sRNA		
						No. of Reads	%	No. of reads	%	No. of Reads	%	
21-nt	700,131	109,114	57,707	166,821	162,197	25,278	15.6	13,369	8.2	38,647	23.8	1.9
22-nt	839,033	251,128	45,522	296,650	194,376	58,178	29.9	10,546	5.4	68,724	35.4	5.5
23-nt	622,373	10,984	3511	14,495	144,183	2545	1.8	813	0.6	3358	2.3	3.1
24-nt	2,155,006	20,556	6561	27,117	499,243	4762	1.0	1520	0.3	6282	1.3	3.1
21–24-nt	4,316,543	391,782	113,301	505,083	1,000,000	90,763	9.1	26,248	2.6	117,011	11.7	3.5

**Table 4:** Summary of the sRNAs identified by NGS from PSTVd-RG1-inoculated tomato plants cv. Rutgers at zero mismatch with the PSTVd-RG1 genome.

sRNA Length	Total sRNA in the Pool	Tolerance: 0			Normalized Reads (Per Million Reads)							(+):(−) vd-sRNA
		(+) vd-sRNA	(−) vd-sRNA	Total vd-sRNA	Total sRNA	(+) vd-sRNA		(−) vd-sRNA		total vd-sRNA		
						No. of Reads	%	No. of Reads	%	No. of Reads	%	
21-nt	160,262	35,533	5778	41,311	219,387	48,642	22.2	7910	3.6	56,552	25.8	6.1
22-nt	145,487	24,528	3913	28,441	199,161	33,577	16.9	5357	2.7	38,934	19.5	6.3
23-nt	124,625	1448	197	1645	170,603	1982	1.2	270	0.2	2252	1.3	7.4
24-nt	300,125	3135	374	3509	410,849	4292	1.0	512	0.1	4804	1.1	8.4
21–24-nt	730,499	64,644	10,272	74,906	1,000,000	88,493	8.8	14,062	1.4	102,555	10.3	6.3

**Table 5:** Summary of the sRNAs identified by NGS from PSTVd-RG1-inoculated tomato plants cv. Rutgers at one mismatch with the PSTVd-RG1 genome.

sRNA Length	Total sRNA in the Pool	Tolerance: 1			Normalized Reads (Per Million Reads)							(+):(−) vd-sRNA
		(+) vd-sRNA	(−) vd-sRNA	Total vd-sRNA	Total sRNA	(+) vd-sRNA		(−) vd-sRNA		total vd-sRNA		
						No. of Reads	%	No. of Reads	%	No. of Reads	%	
21-nt	160,262	46,752	7461	54,213	219,387	64,000	29.2	10,214	4.7	74,214	33.8	6.3
22-nt	145,487	32,779	5121	37,900	199,161	44,872	22.5	7010	3.5	51,882	26.1	6.4
23-nt	124,625	2049	280	2329	170,603	2805	1.6	383	0.2	3188	1.9	7.3
24-nt	300,125	4162	491	4653	410,849	5697	1.4	672	0.2	6370	1.6	8.5
21–24-nt	730,499	85,742	13,353	99,095	1,000,000	117,375	11.7	18,279	1.8	135,654	13.6	6.4

**Table 6:** The runtime of the sRNA profiler on a single core of 3.2 GHz.

ze of the sRNA Pool	Size of the Viroid Genome	Tolerance	Runtime in Seconds
5,875,050	359	0	1450
5,875,050	359	1	1800
730,499	359	0	128
730,499	359	1	131

## Data Availability

All relevant data are within the manuscript and in its Supplementary materials.

## Supplementary Materials

The following are available online at https://www.mdpi.com/article/10.3390/cells10071771/s1. Figure S1: Data generated from the sRNA profiler, showing the details of the sRNA sequences and their occurrence in the pool with reference to circular RNA. Note that if a single sRNA shows matching in more than one place on the genome sequence, it is then counted as the number of times it showed matching with the genome sequence. Figure S2: Data generated from the sRNA profiler, showing the details of the sRNA sequences and their occurrence in the pool with reference to linear RNA. Note that if a single sRNA shows matching in more than one place on the genome sequence, it is then counted as the number of times it showed matching with the genome sequence.
