# Supplementary material for: sRNA Profiler: A User-Focused Interface for Small RNA Mapping and Profiling
Source: Cells. 2021 Jul 13;10(7):1771. doi: 10.3390/cells10071771 (PMC8303536; doi:10.3390/cells10071771)
Supplement: Supplementary file 1 [file cells-10-01771-s001.zip › cells-1269231-supplementary.pdf]

```
Forward matchings

Gene: example_gene.txt
Pool: example_pool.txt
Mismatches: 0
*************************

Length: 5
  Sequence   | Frequency
-------------------------
   GGGAT     |    3
   ATCCC     |    2
   AAACC     |    5
*************************

Length: 6
  Sequence   | Frequency
-------------------------
   TTCAGG    |    1
   CGGGGA    |    3
   CGGCTT    |    1
*************************
```

```
Reverse matchings

Gene: example_gene.txt
Pool: example_pool.txt
Mismatches: 0
*************************

Length: 5
  Sequence   | Frequency
-------------------------
   ATCCC     |    2
   GGGAT     |    3
   TTCCC     |    1
   CGACC     |    2
   AGCCG     |    1
   AAGCC     |    2
*************************

Length: 6
  Sequence   | Frequency
-------------------------
   CCTGAA    |    1
   CGGGGA    |    3
   CAGGTT    |    1
   AGCCGA    |    1
*************************
```

```
Forward matchings

Gene: example_gene.txt
Pool: example_pool.txt
Mismatches: 1
*************************

Length: 5
  Sequence   | Frequency
-------------------------
   TCAGG     |    1
   GGGAT     |    5
   GATCC     |    1
   ATCCC     |    8
   TCCCC     |    1
   GGGAA     |    4
   GGAAA     |    2
   GAAAC     |    2
   AAACC     |    7
   TCGGC     |    1
*************************

Length: 6
  Sequence   | Frequency
-------------------------
   TTCAGG    |    2
   CAGGGA    |    3
   CGGGGA    |    4
   GGGGAA    |    1
   CGGCTT    |    2
*************************
```

```
Reverse matchings

Gene: example_gene.txt
Pool: example_pool.txt
Mismatches: 1
*************************

Length: 5
  Sequence   | Frequency
-------------------------
   CTGAA     |    1
   ATCCC     |    8
   GATCC     |    1
   GGGAT     |    5
   TCCCC     |    1
   TTCCC     |    4
   TTTCC     |    2
   GTTTC     |    1
   AGGTT     |    1
   CGACC     |    4
   AGCCG     |    5
   AAGCC     |    8
*************************

Length: 6
  Sequence   | Frequency
-------------------------
   CCTGAA    |    1
   CCCTGA    |    1
   CGGGGA    |    4
   AGGTTT    |    1
   CAGGTT    |    1
   AGCCGA    |    2
*************************
```

Figure S1 (Adkar-Purushothama et al.,)

```
Forward matchings

Gene: example_gene.txt
Pool: example_pool.txt
Mismatches: 0
************************

Length: 5
 Sequence  | Frequency
-------------------------
  GGGAT   |   3
  ATCCC   |   2
  AAACC   |   5
************************

Length: 6
 Sequence  | Frequency
-------------------------
  TTCAGG   |   1
  CGGGGA   |   3
************************
```

```
Reverse matchings

Gene: example_gene.txt
Pool: example_pool.txt
Mismatches: 0
************************

Length: 5
 Sequence  | Frequency
-------------------------
  ATCCC   |   2
  GGGAT   |   3
  TTCCC   |   1
  CGACC   |   2
************************

Length: 6
 Sequence  | Frequency
-------------------------
  CCTGAA   |   1
  CGGGGA   |   3
  CAGGTT   |   1
************************
```

```
Forward matchings

Gene: example_gene.txt
Pool: example_pool.txt
Mismatches: 1
************************

Length: 5
 Sequence  | Frequency
-------------------------
  TCAGG   |   1
  GGGAT   |   5
  GATCC   |   1
  ATCCC   |   8
  TCCCC   |   1
  GGGAA   |   4
  GGAAA   |   2
  GAAAC   |   2
  AAACC   |   7
************************

Length: 6
 Sequence  | Frequency
-------------------------
  TTCAGG   |   2
  CAGGGA   |   3
  CGGGGA   |   4
  GGGGAA   |   1
************************
```

```
Reverse matchings

Gene: example_gene.txt
Pool: example_pool.txt
Mismatches: 1
************************

Length: 5
 Sequence  | Frequency
-------------------------
  CTGAA   |   1
  ATCCC   |   8
  GATCC   |   1
  GGGAT   |   5
  TCCCC   |   1
  TTCCC   |   4
  TTTCC   |   2
  GTTTC   |   1
  AGGTT   |   1
  CGACC   |   4
************************

Length: 6
 Sequence  | Frequency
-------------------------
  CCTGAA   |   1
  CCCTGA   |   1
  CGGGGA   |   4
  AGGTTT   |   1
  CAGGTT   |   1
************************
```

Figure S2 (Adkar-Purushothama et al.,)
